# Construction of TiO_2_/CuPc Heterojunctions for the Efficient Photocatalytic Reduction of CO_2_ with Water

**DOI:** 10.3390/molecules29081899

**Published:** 2024-04-22

**Authors:** Jun Wang, Shuang Fu, Peng Hou, Jun Liu, Chao Li, Hongguang Zhang, Guowei Wang

**Affiliations:** 1 Academic Affairs Office, Qiqihar Medical University, Qiqihar 161006, China; 18845216002@163.com; 2 College of Pharmacy, Qiqihar Medical University, Qiqihar 161006, China; fsjt1980@qmu.edu.cn (S.F.); houp@qmu.edu.cn (P.H.); liuj@qmu.edu.cn (J.L.); lichao@qmu.edu.cn (C.L.); 3 College of Pathology, Qiqihar Medical University, Qiqihar 161006, China

**Keywords:** TiO_2_/CuPc, heterojunction, photocatalysis, charge separation, CO_2_ reduction

## Abstract

Utilizing solar energy for photocatalytic CO_2_ reduction is an attractive research field because of its convenience, safety, and practicality. The selection of an appropriate photocatalyst is the key to achieve efficient CO_2_ reduction. Herein, we report the synthesis of TiO_2_/CuPc heterojunctions by compositing CuPc with TiO_2_ microspheres via a hydroxyl-induced self-assembly process. The experimental investigations demonstrated that the optimal TiO_2_/0.5CuPc photocatalyst exhibited a significantly enhanced CO_2_ photoreduction rate up to 32.4 μmol·g^−1^·h^−1^ under 300 W xenon lamp irradiation, which was 3.7 times that of the TiO_2_ microspheres alone. The results of photoelectrochemical experiments indicated that the construction of the heterojunctions by introducing CuPc effectively promoted the separation and transport of photogenerated carriers, thus enhancing the catalytic effect of the photocatalyst.

## 1. Introduction

The extensive exploitation of fossil fuels has dramatically increased the amount of carbon dioxide (CO_2_) in the atmosphere, which has led to a global greenhouse effect that is worsening year by year and poses a serious threat to the survival of humankind [1,2]. Photocatalytic CO_2_ reduction technology is an ideal way to mitigate the greenhouse effect due to its advantages, including mild operating conditions, low energy consumption, and the absence of secondary pollution [3,4,5]. The characteristics of the photocatalyst are generally considered to be among the most important factors determining the efficiency of photocatalytic CO_2_ conversion. The development of an effective photocatalyst has therefore gained continuous attention.

Currently, various photocatalytic materials with enhanced CO_2_ conversion effects have been developed [6,7,8,9,10]. Among them, titanium dioxide (TiO_2_), a promising semiconductor material, has a wide range of applications in photocatalysis due to its unusual electronic and optical properties [11,12,13,14]. For this reason, it has received extensive research and attention. However, it still suffers a low CO_2_ reduction efficiency, primarily due to the photogenerated electron-hole pairs being prone to recombination and the substantially wide bandgap (3.2 eV). So far, various strategies have been developed to enhance the photocatalytic performance of TiO_2_ for CO_2_ reduction, including the introduction of surface defects [15], the doping of heteroatoms [16], and the construction of heterojunctions [17]. Previous studies have successfully demonstrated that the construction of heterojunctions with narrow bandgap semiconductors is a reliable way to improve the photocatalytic activity of TiO_2_ [18,19]. For example, Ejaz Hussain et al. synthesized Au@TiO_2_/CdS hybrid catalysts through hydrothermal reactions and found that Au@TiO_2_/CdS was the most active catalyst, producing 19.15 mmol·g^−1^·h^−1^ of hydrogen under sunlight [20]. Dai et al. successfully prepared TiO_2_/CuS nanocomposites with cauliflower-like protrusions using a simple one-step hydrothermal method with the assistance of 3-mercaptopropionic acid (3-MPA) [21]. Their experimental results showed that the TiO_2_/CuS nanocomposites exhibited a better photocatalytic performance compared to TiO_2_ and CuS controls. Yin et al. synthesized visible-light-responsive Ag_3_PO_4_/OH/TiO_2_ catalysts through the in situ growth of Ag_3_PO_4_ on the surface of TiO_2_ with alkali treatment [22]. The introduction of Ag_3_PO_4_ effectively improved the light absorption ability of the photocatalysts, which enabled the catalysts to achieve a 90% degradation of RhB under visible light. Although the above approaches effectively improved the photocatalytic activity of TiO_2_, it is still inefficient in photocatalytic CO_2_ reduction because of its lack of catalytic sites.

Very recently, several studies have found that metal phthalocyanines (MPcs) can be used to construct efficient heterojunction photocatalysts with TiO_2_ due to their suitable energy band structure and metal active center unit [23,24]. On the one hand, the porphyrin rings in metal phthalocyanines, analogous to chlorophylls, are widely used as photosensitizers to effectively improve the light absorption of photocatalysts. On the other hand, the central metal of metal phthalocyanines can provide efficient active sites for photocatalytic CO_2_ reduction. For example, Altuğ Mert Sevim found that the photocatalytic degradation performance of a composite photocatalyst for 4-chlorophenol under visible-light irradiation was greatly enhanced through the introduction of metal phthalocyanine into TiO_2_ [25]. It was also reported by Fei that FePc/TiO_2_ catalysts demonstrated good photocatalytic activity for the degradation of organic contaminants [26]. Makoto Endo reported the synthesis of ZnPc/TiO_2_ hybrid nanomaterials and evaluated their photocatalytic reduction of CO_2_ [27]. It was found that modification of the TiO_2_ with ZnPc could indeed improve its CO_2_ photoconversion performance. The above examples successfully suggest that the construction of heterojunctions using metal phthalocyanines and TiO_2_ for the efficient conversion of CO_2_ is a reasonable design.

In this work, we successfully synthesized a series of TiO_2_ microspheres loaded with different amounts of CuPc. The unique selective absorption for CuPc in the range of 500~800 nm can effectively solve the defect of poor visible-light utilization of TiO_2_, resulting in heterojunctions with enhanced light absorption capabilities. It was found that the developed TiO_2_/0.5CuPc photocatalyst exhibited increased CO_2_ reduction activity compared to pristine TiO_2_. This enhancement of the photoactivity was attributed to the construction of heterojunctions, which promote the efficient separation of photogenerated charges, as demonstrated in the photoelectrochemical experiments. Moreover, the photocatalytic CO_2_ conversion process was investigated through in situ diffuse reflectance infrared Fourier-transform spectroscopy (DRIFTS).

## 2. Results and Discussion

### 2.1. Catalyst Characterization

**Figure 1:**
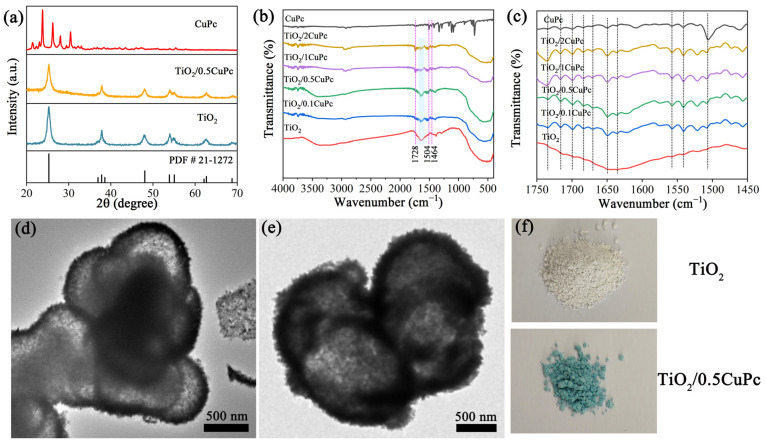
(**a**) XRD patterns of TiO_2_, CuPc, and TiO_2_/0.5CuPc. FTIR spectra at (**b**) 400–4000 cm^−1^ and (**c**) 1450–1750 cm^−1^ of TiO_2_, CuPc, and TiO_2_/xCuPc. TEM images of (**d**) TiO_2_ and (**e**) TiO_2_/0.5CuPc. (**f**) Photographs of TiO_2_ and TiO_2_/0.5CuPc.

The crystal structure and composition of the as-synthesized samples were investigated using X-ray diffraction patterns (XRD). Typical XRD patterns depict the crystal structures of pure TiO_2_, CuPc, and TiO_2_/0.5CuPc composites in Figure 1a. The TiO_2_ sample exhibited seven characteristic diffraction peaks at 25.3°, 37.9°, 48.0°, 54.1°, 55.1°, 62.8°, and 68.7°, assigned to the (101), (004), (200), (105), (211), (204), and (116) crystal planes, respectively. These diffraction peaks could be indexed to the anatase TiO_2_ crystal structure (JCPDS NO. 21-1272). In addition, it can be seen that the intensity of the characteristic diffraction peaks of TiO_2_ was slightly reduced after the introduction of CuPc, which might be attributed to the fact that the characteristic peaks of TiO_2_ were suppressed by the coated CuPc [28,29]. However, no significant new peaks attributed to CuPc appeared in the XRD spectrum of TiO_2_/0.5CuPc compared to that of TiO_2_, indicating the low loading content of CuPc. The chemical structures of TiO_2_ and TiO_2_/xCuPc were further analyzed using FTIR spectroscopy. As shown in Figure 1b, the broader absorption peak located in the range of 400–800 cm^−1^ can be attributed to the stretching vibration of Ti-O and Ti-O-Ti [30]. In contrast, the successful loading of CuPc onto TiO_2_ can be identified by the characteristic peaks (1464, 1504, and 1728 cm^−1^), which correspond to the phthalocyanine backbone and the central metal and ligand of CuPc [31]. As shown in Figure 1c, it was obvious that multiple peaks corresponding to the phthalocyanine backbone vibrations appeared in the TiO_2_ samples after modification with CuPc. In addition, the intensity of the vibrational peak of the TiO_2_ surface hydroxyl group located at 1640 cm^−1^ was significantly weaker after CuPc modification (the marked area), suggesting that CuPc interacted with the surface hydroxyl group (Figure 1b). The TEM images of the TiO_2_ and TiO_2_/0.5CuPc heterojunction are shown in Figure 1d,e. Obviously, the TiO_2_ exhibited a spherical structure with a partially hollow core (Figure 1d). Many microspheres were clustered together and therefore exhibited poor dispersibility. It can be seen from Figure 1e that the CuPc modification did not affect the morphology of the TiO_2_ microspheres and uniformly covered the surface of the TiO_2_. In addition, as can be seen from Figure 1f, the color of the TiO_2_ sample changed from light yellow to blue after loading with CuPc, indicating that the CuPc had been successfully loaded onto the surface of the TiO_2_.

**Figure 2:**
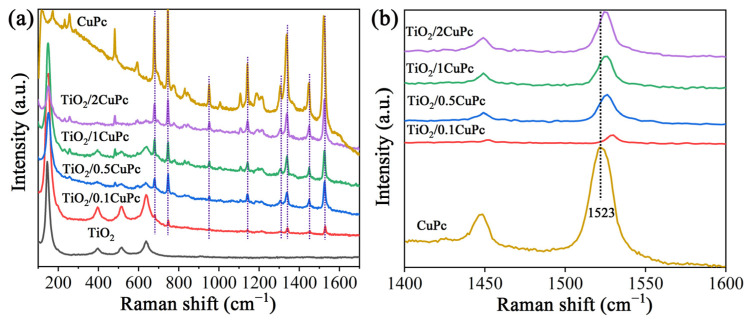
(**a**) Raman spectra and (**b**) partially magnified Raman spectra of TiO_2_, CuPc, and TiO_2_/xCuPc.

Raman spectroscopy was utilized to further investigate the structures of TiO_2_ and TiO_2_/xCuPc. As expected, the Raman spectra prove that the TiO_2_ microspheres exhibited an anatase phase (Figure 2a). The characteristic peaks at 146, 396, 516, and 637 cm^−1^ are assigned to the E_g(1)_, B_1g_, A_1g_, and E_g(3)_ lattice vibration modes of the anatase phase, respectively [32]. The Raman spectra of TiO_2_/xCuPc show both TiO_2_ and CuPc characteristic peaks, indicating that the TiO_2_/xCuPc heterojunctions were successfully synthesized. It was observed that the intensity of the characteristic peaks of CuPc gradually increased with the increase in the amount of CuPc modification, while the intensity of the characteristic peaks of TiO_2_ gradually decreased. In addition, the Raman vibration peak at 1523 cm^−1^ (the tensile of C-N-C bridge bonds in the CuPc) was shifted towards the long-wave-number direction after the formation of TiO_2_/xCuPc heterojunctions, which may have been due to the occurrence of a self-assembly of CuPc on the TiO_2_ surface (Figure 2b) [33,34].

**Figure 3:**
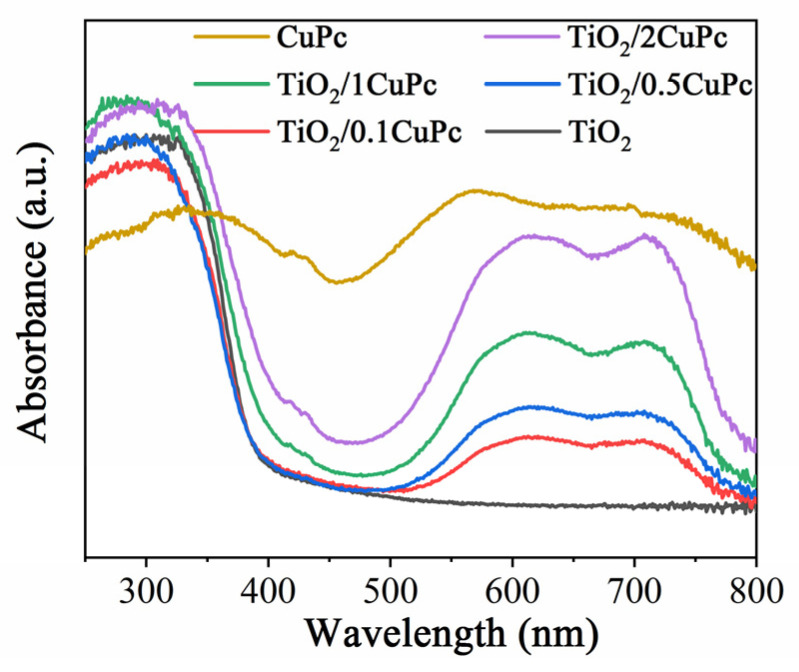
UV-vis DRS reflectance spectra of TiO_2_, CuPc, and TiO_2_/xCuPc.

Figure 3 shows the UV-Vis DRS reflectance spectra of TiO_2_, CuPc, and TiO_2_/xCuPc. The absorption edge of the TiO_2_ sample was observed at approximately 420 nm. However, the TiO_2_/xCuPc heterojunctions exhibited strong light absorption in the visible region of 500~800 nm, which can be attributed to the resulting Q-band electron transition of CuPc from its highest occupied molecular orbital (HOMO) to its lowest unoccupied molecular orbital (LUMO) [35]. In addition, it can also be seen that the absorption intensity gradually increased as the amount of CuPc increased.

**Figure 4:**
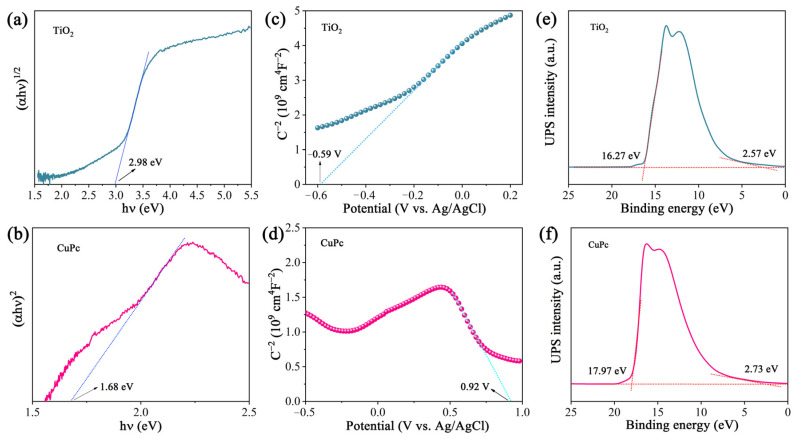
(**a**) Plot of (αhv)^1/2^ versus (hv) for the band gap energy of TiO_2_ and (**b**) plot of (αhv)^2^ versus (hv) for the band gap energy of CuPc. Mott–Schottky plots of (**c**) TiO_2_ and (**d**) CuPc. UPS spectra of (**e**) TiO_2_ and (**f**) CuPc.

The optical band gap of a catalyst can be calculated from its light absorption spectra according to the equation *αhv* = *A*(*hv* − *E_g_*)*^n/2^*, where *α*, *h*, *ν*, *A*, and *E_g_* represent the absorption coefficient, Planck constant, light frequency, proportionality, and band gap energy, respectively. For TiO_2_ and CuPc, the values of n are 1 and 4, respectively. Based on the above equation, the calculated *E_g_* values for TiO_2_ and CuPc are 2.98 and 1.68 eV, respectively (Figure 4a,b). To further investigate the band structures of TiO_2_ and CuPc, Mott–Schottky curves were obtained. A positive slope of the Mott–Schottky plot would indicate that TiO_2_ is an n-type semiconductor, while a negative slope of the Mott–Schottky plot would indicate that CuPc is a p-type semiconductor. As shown in Figure 4c,d, the flat band potentials of TiO_2_ and CuPc were determined to be −0.59 V and 0.92 V vs. Ag/AgCl, respectively. According to the formula E(RHE) = E(Ag/AgCl) + 0.197 + 0.059 pH, we could deduce that the conduction band potential (E_CB_) of TiO_2_ and the highest occupied molecular orbital (HOMO) energy level of CuPc were approximately 0.01 and 1.52 V vs. RHE, respectively. According to the empirical equation E_g_ = E_VB_ − E_CB_, the valence band potential (E_VB_) of TiO_2_ and the lowest unoccupied molecular orbital (LUMO) energy level of CuPc were calculated to be 2.99 and −0.16 V vs. RHE, respectively. In addition, ultraviolet photoelectron spectroscopy (UPS) was also performed to determine the valence band energy (E_VB_) of TiO_2_ and CuPc (Figure 4e,f). The incident photon energy (hv) of the helium I source was 21.22 eV [36,37]. By subtracting the width of the peak from the excitation energy (21.22 eV), the valence band maximum of TiO_2_ and the HOMO energy of CuPc were calculated to be 7.52 and 5.98 eV, respectively, on the absolute vacuum scale (AVS). According to the reference standard, 0 V for the reversible hydrogen electrode (RHE) is equal to 4.44 eV on the vacuum level. Therefore, the E_VB_ (versus RHE) value of TiO_2_ and the HOMO energy of CuPc were calculated to be 3.08 and 1.54 V, respectively. That is, the conduction band energy (E_CB_) of TiO_2_ and the LUMO energy of CuPc were 0.10 and −0.14 V, respectively. These results are consistent with those found in the Mott–Schottky plot calculations.

**Figure 5:**
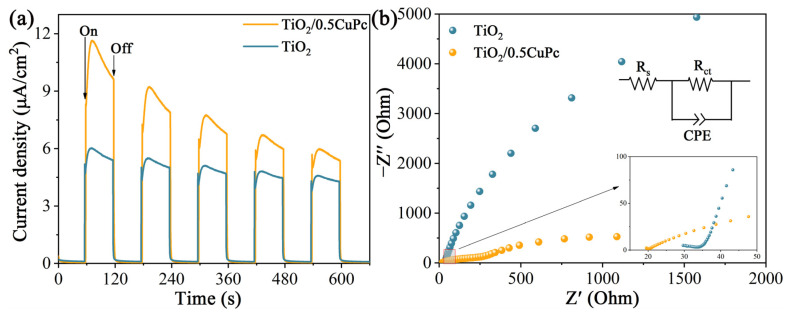
(**a**) Transient photocurrent responses and (**b**) EIS Nyquist plots of TiO_2_ and TiO_2_/0.5CuPc.

Photoelectrochemical experiments were applied to reveal the charge transfer kinetics of the heterojunctions. As shown in Figure 5a, the photocurrent density of TiO_2_/0.5CuPc was higher than that of TiO_2_, which indicates that the formation of a heterojunction can indeed effectively inhibit the recombination and further promote the separation of photogeneration carriers. The separation efficiency of photogeneration charges in the heterojunctions was further verified using the impedance spectroscopy spectra (EIS) (Figure 5b). It is obvious that the arc radius in the Nyquist plot of TiO_2_/0.5CuPc is much smaller than that of the TiO_2_ plot, suggesting that the charge transfer resistance in the heterojunction was reduced and facilitated rapid carrier separation and transfer. As shown in the inset of Figure 5b, the arc radius of TiO_2_/0.5CuPc was similarly smaller than TiO_2_ in the high-frequency region, suggesting better conductivity and proving the lower recombination rate of the carriers.

### 2.2. Photocatalytic Performance and Reaction Mechanism

**Figure 6:**
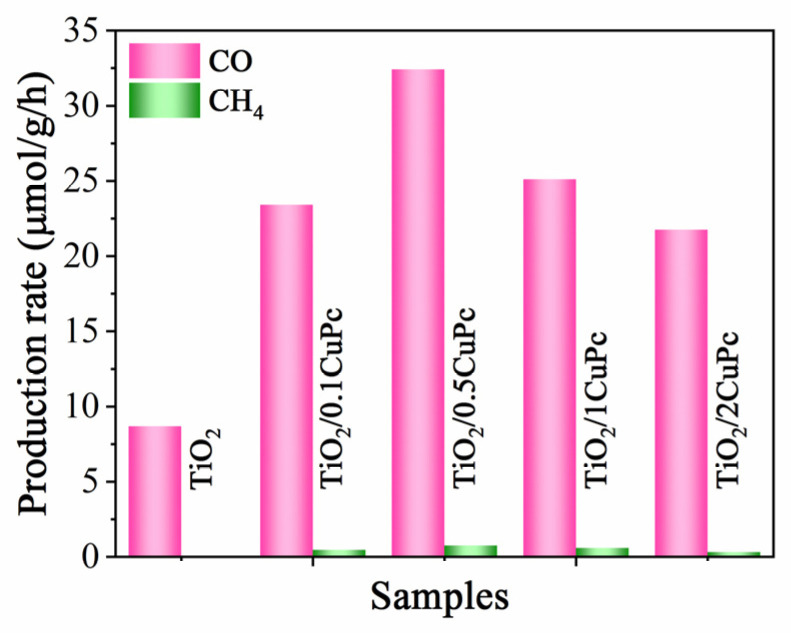
Photocatalytic activities for CO_2_ reduction of the TiO_2_ and TiO_2_/0.5CuPc heterojunction.

The photocatalytic CO_2_ reduction performance of the TiO_2_ and TiO_2_/xCoPc was tested under 300 W Xe lamp illumination. As shown in Figure 6, the reduction products CH_4_ and CO were detected. The sample of TiO_2_ exhibited a low CO_2_ reduction activity with a production rate of 8.7 μmol·g^−1^·h^−1^ for CO. However, integration with CuPc significantly enhanced the photocatalytic activity of TiO_2_. It was noticed that the photocatalytic performance of the TiO_2_/xCoPc heterojunctions first increased and then decreased with the increase in CuPc loading. The higher the CuPc loading, the more severe the agglomeration of CuPc units at the TiO_2_ surface, thus leading to a decrease in photocatalytic activity [38]. The maximum CO production performance rate of 32.4 μmol·g^−1^·h^−1^ was attained with the TiO_2_/0.5CoPc heterojunction, which was 3.7 times higher than that of the TiO_2_ microspheres. In addition, the yield of CH_4_ was almost negligible, which indicates that the TiO_2_/0.5CoPc heterojunction has high selectivity for CO_2_ reduction to CO.

**Figure 7:**
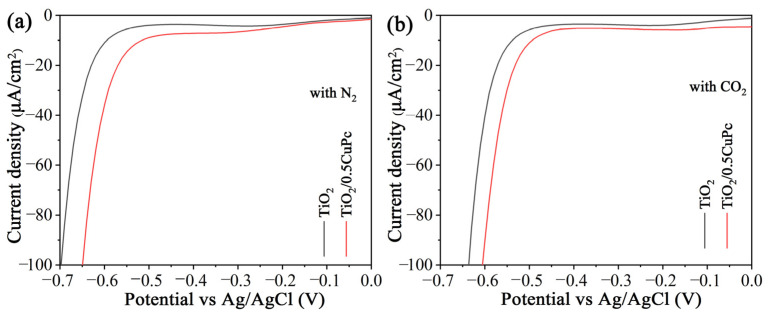
Electrochemical reduction curves of the TiO_2_ and TiO_2_/0.5CuPc heterojunction in (**a**) a N_2_-bubbled system and (**b**) a CO_2_-bubbled system, respectively.

The mechanism of the CO_2_ reduction process was investigated using electrochemical reduction measurements in different gas-bubbled systems. As shown in Figure 7a,b, it was obvious that the onset potential of TiO_2_/0.5CoPc heterojunction was lower than that of the TiO_2_ microspheres in both N_2_-saturated and CO_2_-saturated electrolytes. Furthermore, the onset potential of the heterojunction in the CO_2_-saturated electrolyte was lower than that in the N_2_-saturated electrolyte, suggesting that the CuPc modification was more favorable for CO_2_ activation [39].

**Figure 8:**
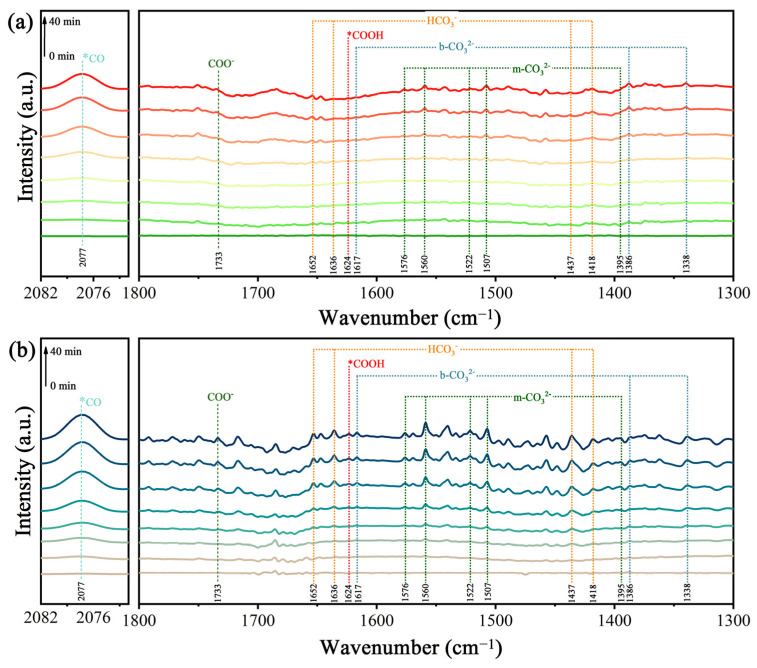
The in situ DRIFT spectra of (**a**) TiO_2_ and (**b**) the TiO_2_/0.5CuPc heterojunction at different light irradiation intervals.

The intermediates in CO_2_ conversion were explored using in situ diffuse reflectance infrared Fourier-transform spectroscopy (Figure 8). The absorption peaks located at 1338, 1386, and 1617 cm^−1^ are ascribed to bidentate carbonates (b-CO_3_^2−^), and the peaks at 1395, 1507, 1522, 1560, and 1576 cm^−1^ belong to monodentate carbonate (m-CO_3_^2−^) [40]. Bands at 1418, 1437, 1636, and 1652 cm^−1^ can also be observed, which can be assigned to the HCO_3_^−^ groups. The absorption peak at 2077 cm^−1^ is attributable to CO. In addition, with the increase in irradiation time, it can be seen that a peak of the COO^−^ radical at 1624 cm^−1^ and a peak of COOH* at 1733 cm^−1^ began to appear [41], and the peak intensity increased gradually. These results indicate that this photocatalytic reaction for the reduction of CO_2_ was carried out efficiently. It is worth noting that the formation of the important intermediate COOH* is generally considered to be a rate-limiting step during the photocatalytic conversion of CO_2_ to CO. It is obvious that the peak intensity of COOH* in the TiO_2_/0.5CoPc heterojunction was stronger compared to that of TiO_2_ at the same light irradiation time, which indicates the better activity of the TiO_2_/0.5CoPc heterojunction for the photocatalytic conversion of CO_2_.

**Figure 9:**
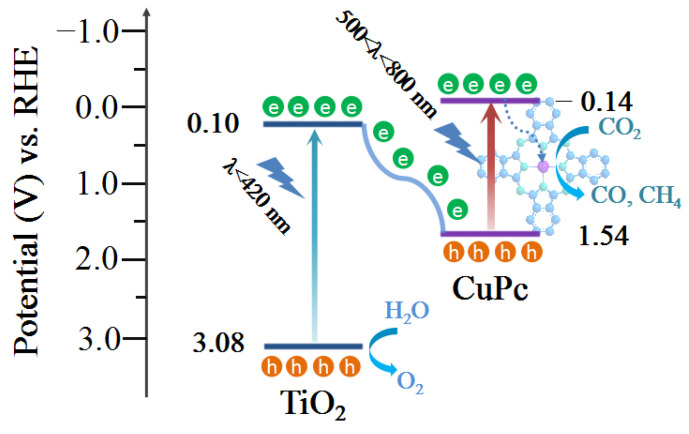
Schematic illustration of the proposed photocatalytic CO_2_ reduction mechanism of the TiO_2_/0.5CuPc heterojunction.

Based on the above discussions, a mechanism of charge transfer and separation to promote CO_2_ conversion is proposed (Figure 9). First, the TiO_2_ and CuPc absorbed enough light of different wavelengths for electron transition under the 300 W Xe lamp irradiation to generate photogenerated carriers (e^−^/h^+^ pairs). Because of the well-matched energy levels of TiO_2_ and CuPc, the photogenerated electrons generated through the excitation of TiO_2_ were transferred to the HOMO energy level of CuPc and recombined with the holes of CuPc. As a result, the remaining holes in the TiO_2_ valence band could be used for the oxidation of H_2_O to O_2_, while the separated electrons in the LUMO energy level of CuPc were transferred to coordinated central metal ions for the CO_2_ reduction reaction. Based on the in situ diffuse reflectance infrared Fourier-transform spectroscopy (DRIFTS) results, the possible CO_2_ reduction pathways are as follows:

CO_2_ (g) → CO_2_* CO_2_ + H^+^ + e^−^ → COOH* COOH* + H^+^ + e^−^ → CO* + H_2_O CO* → CO

## 3. Materials and Methods

### 3.1. Materials

All of the chemical reagents were purchased from Aladdin Chemical Reagents Limited and were of analytical grade and used without further purification: titanium sulfate (Ti(SO_4_)_2_, 99%), ethylenediaminetetraacetic acid (EDTA, 99.5%), copper(II) phthalocyanine (CuPc, 97%), ethanol (C_2_H_5_OH, 99.99%), and sodium sulfate anhydrous (Na_2_SO_4_, 99%). Deionized water was used throughout.

### 3.2. Synthesis of TiO_2_ Microspheres

The TiO_2_ microspheres were synthesized through a simple hydrothermal method. At first, Ti(SO_4_)_2_ (0.2400 g, 1 mmol) and EDTA (1.4612 g, 5 mmol) were dissolved in 30 mL of deionized water under stirring. After that, the solution was transferred into an autoclave and was treated at 180 °C for 8 h in a temperature-controlled oven. The resulting product was filtered and washed with deionized water. Finally, the obtained solid was dried in an oven at 60 °C overnight. 

### 3.3. Synthesis of TiO_2_/CuPc Heterojunction

The TiO_2_/CuPc heterojunctions were prepared using a hydroxyl-induced self-assembly process based on the reported method [35]. In a typical experiment, 40 mg of TiO_2_ microspheres was dispersed in 25 mL of ethanol and sonicated for 30 min, noted as Solution A. Various amounts of CuPc powder were then dispersed in 25 mL of ethanol and sonicated for 30 min, noted as Solution B. Solution A and B were then mixed and sonicated for another 30 min. Afterwards, the above solution was evaporated in a water bath at 75 °C under magnetic stirring. After drying at 80 °C for 4 h in an oven, TiO_2_/xCuPc heterojunctions (where x = 0.1, 0.5, 1, or 2) were obtained, with x representing the mass ratio percentage of CuPc to TiO_2_. For example, weighing 40 mg of TiO_2_ microspheres required the addition of 0.2 mg of CuPc, resulting in a mass percentage of CuPc to TiO_2_ of 0.5%, noted as TiO_2_/0.5CuPc. Similarly, if 0.04 mg, 0.4 mg, and 0.8 mg amounts of CuPc were added, respectively, we obtained TiO_2_/0.1CuPc, TiO_2_/1CuPc, and TiO_2_/2CuPc.

### 3.4. Characterization

The morphology and structure of each samples were characterized by using transmission electron microscopy (JEOL, JEM-F200, Tokyo, Japan) with an acceleration voltage of 200 kV. X-ray powder diffraction analysis was recorded under ambient conditions with a Shimadzu XRD-6000 diffractor (Kyoto, Japan) with Cu K radiation (0.15405 nm) at 40 kV and 40 mA. Raman spectra of the samples were measured using a Renishaw inVia Reflex spectrometer system (λ = 532 nm) (London, UK). Fourier-transform infrared (FT-IR) spectra were recorded using a Thermo Scientific Nicolet iS50 (Waltham, MA, USA), with KBr as the diluent. UV-vis absorption spectra were recorded with a Shimadzu UV2700 spectrophotometer (Kyoto, Japan), using BaSO_4_ as the reference. The electrochemical studies were detected on an H-type cell using an electrochemical workstation (IVIUM V13806, Amsterdam, The Netherlands). Ultraviolet photoelectron spectroscopy (UPS) measurements were performed on ESCALAB 250Xi (Waltham, MA, USA) with an unfiltered HeI (21.22 eV) gas discharge lamp and a total instrumental energy resolution of 100 meV. In situ diffuse reflectance infrared Fourier-transform spectroscopy (DRIFTS) measurements were performed by using the Nicolet iS50 Fourier-transform spectrometer (Waltham, MA, USA) equipped with an MCT diffuse reflectance accessory.

### 3.5. Photocatalytic CO_2_ Reduction

Photocatalytic CO_2_ reduction was conducted in a 100 mL quartz cell reactor equipped with a 300 W xenon lamp (PLSSXE300UV, PerfectLight, Beijing, China) as the light source. In detail, 10 mg of the photocatalyst and 10 mL of deionized water were added to the 100 mL quartz cell reactor. High-purity CO_2_ gas (99.9%) was passed through the water and then into the reaction setup to reach an ambient pressure. The photocatalysts were allowed to equilibrate in the CO_2_/H_2_O system for 20 min under stirring, and were then irradiated with the 300 W xenon lamp. The amounts of CO and CH_4_ that evolved were determined using a gas chromatograph (Techcomp GC-7900, Shanghai, China) equipped with both TCD and FID detectors. The production rates of CO and CH_4_ were calculated according to the standard curve.

### 3.6. Photoelectrochemical Measurements

The film electrode was fabricated as follows: firstly, 10 mg of the sample, 0.1 mL of Nafion, and 0.9 mL of ethanol were mixed into a slurry thoroughly. Then, the slurry was coated onto the FTO glass electrode (1.0 cm × 1.0 cm). Lastly, the coated electrode was dried at 60 °C for 30 min. Photoelectrochemical (PEC) measurements were carried out using the IVIUM V13806 electrochemical workstation with a traditional three-electrode system. The as-prepared sample films were used as working electrodes in the sealed quartz cell. A platinum plate (99.9%) and a saturated KCl Ag/AgCl electrode were used as the counter electrode and reference electrode, respectively. A 0.2 mol·L^−1^ Na_2_SO_4_ solution was used as the electrolyte (pH = 6.8). PEC experiments were performed in a quartz cell using a 300 W xenon lamp as the illumination source. Mott–Schottky plots were implemented at frequencies of 1000 Hz. All of the experiments were performed at room temperature (about 25 ± 3 °C).

### 3.7. Electrochemical Reduction Measurements

Electrochemical reduction measurements were carried out in a traditional three-electrode system. The working electrode was a 0.3 cm diameter glassy carbon (GC) electrode, a saturated KCl Ag/AgCl electrode was used as the reference electrode, and a Pt sheet was used as the counter electrode. Five milligrams of each different sample mixed with 20 μL of a 5 wt % Nafion ionomer was dissolved in 0.18 mL of an aqueous ethanol solution. The catalyst ink was scanned with ultrasound for 30 min, and a suitable mass of the ink was uniformly dropped onto the clean GC electrode surface and dried in air. An IVIUM V13806 electrochemical workstation was employed to test the electrochemical activity and stability of the series of catalysts. High-purity N_2_ or CO_2_ (99.999%) were employed to bubble through the electrolyte to keep the gas saturated in the EC experiment. At the beginning, electrode potentials were cycled between two potential limits until perfectly overlapping; afterward, the I–V curves were obtained. For the electrolytes in the tests, 1 mol·L^−1^ Na_2_SO_4_ was used. The scan rate of the linear sweep voltammetry was 50 mV/s. All of the experiments were performed at room temperature (about 25 ± 3 °C).

### 3.8. In-Situ Diffuse Reflectance Infrared Fourier Transform Spectroscopy (DRIFTS)

In situ DRIFTS measurements were performed using a Nicolet iS50 Fourier-Transform Spectrometer equipped with an MCT diffuse reflectance accessory. Each spectrum was recorded at a resolution of 4 cm^−1^ by averaging 16 scans. The samples were compressed and stored in a custom-fabricated infrared reaction chamber sealed with a ZnSe window. Before measurement, each catalyst was purged with nitrogen at 170 °C for 3 h to remove any surface-adsorbed impurities. The samples were then cooled to room temperature and the background spectra were collected. Subsequently, a mixture of carbon dioxide and water vapor was introduced into the reaction chamber until the adsorption reached equilibrium. The samples were then swept with nitrogen to remove the unadsorbed gases. Subsequently, FT-IR spectra were collected at different irradiation intervals under 300 W xenon lamp irradiation.

## 4. Conclusions

In conclusion, this study successfully demonstrates the construction of TiO_2_/CuPc heterojunctions that significantly enhance CO_2_’s photoreduction to CO. Benefiting from the complementary light-absorbing properties of CuPc and TiO_2_, combined with the superior photogenerated charge separation efficiency, the developed TiO_2_/0.5CuPc photocatalyst exhibited a better photocatalytic CO_2_ reduction performance than that of pristine TiO_2_. In addition, the presence of the metal Cu center in CuPc further acted as a catalytic site, enhancing the CO_2_ reduction process. These findings not only highlight a facile strategy for enhancing TiO_2_ photocatalyst activity but also pave the way for future advancements in photocatalytic technology for environmental remediation and the sustainable conversion of greenhouse gases into valuable resources.

## Data Availability

Data are contained within the article.
